# Prognostic Value of Multiple Circulating Biomarkers for 2-Year Death in Acute Heart Failure With Preserved Ejection Fraction

**DOI:** 10.3389/fcvm.2021.779282

**Published:** 2021-12-09

**Authors:** Yan Gao, Xueke Bai, Jiapeng Lu, Lihua Zhang, Xiaofang Yan, Xinghe Huang, Hao Dai, Yanping Wang, Libo Hou, Siming Wang, Aoxi Tian, Jing Li

**Affiliations:** ^1^National Clinical Research Center for Cardiovascular Diseases, Fuwai Hospital, Chinese Academy of Medical Sciences and Peking Union Medical College, National Center for Cardiovascular Diseases, Beijing, China; ^2^Fuwai Hospital, Chinese Academy of Medical Sciences, Shenzhen, China

**Keywords:** heart failure, preserved ejection fraction, biomarkers, prognostic, risk of death

## Abstract

**Background:** Heart failure with preserved ejection fraction (HFpEF) is increasingly recognized as a major global public health burden and lacks effective risk stratification. We aimed to assess a multi-biomarker model in improving risk prediction in HFpEF.

**Methods:** We analyzed 18 biomarkers from the main pathophysiological domains of HF in 380 patients hospitalized for HFpEF from a prospective cohort. The association between these biomarkers and 2-year risk of all-cause death was assessed by Cox proportional hazards model. Support vector machine (SVM), a supervised machine learning method, was used to develop a prediction model of 2-year all-cause and cardiovascular death using a combination of 18 biomarkers and clinical indicators. The improvement of this model was evaluated by c-statistics, net reclassification improvement (NRI), and integrated discrimination improvement (IDI).

**Results:** The median age of patients was 71-years, and 50.5% were female. Multiple biomarkers independently predicted the 2-year risk of death in Cox regression model, including N-terminal pro B-type brain-type natriuretic peptide (NT-proBNP), high-sensitivity cardiac troponin T (hs-TnT), growth differentiation factor-15 (GDF-15), tumor necrosis factor-α (TNFα), endoglin, and 3 biomarkers of extracellular matrix turnover [tissue inhibitor of metalloproteinases (TIMP)-1, matrix metalloproteinase (MMP)-2, and MMP-9) (FDR < 0.05). The SVM model effectively predicted the 2-year risk of all-cause death in patients with acute HFpEF in training set (AUC 0.834, 95% CI: 0.771–0.895) and validation set (AUC 0.798, 95% CI: 0.719–0.877). The NRI and IDI indicated that the SVM model significantly improved patient classification compared to the reference model in both sets (*p* < 0.05).

**Conclusions:** Multiple circulating biomarkers coupled with an appropriate machine-learning method could effectively predict the risk of long-term mortality in patients with acute HFpEF. It is a promising strategy for improving risk stratification in HFpEF.

## Introduction

Heart failure (HF) is a leading cardiovascular disorder with high morbidity and mortality (1). Based on measurement of left ventricular ejection fraction (LVEF), HF is categorized into heart failure with reduced ejection fraction (HFrEF, LVEF < 40%), HF with preserved ejection fraction (HFpEF, LVEF ≥ 50%), and HF with a mid-range ejection fraction of 40 to 50% (2, 3). HFpEF accounts for nearly half of HF patients worldwide, which is increasingly recognized as a major challenge for clinical practice due to no effective management and pharmacological interventions (2–4). Therefore, accurate risk stratification is critical for tailoring treatment and long-term management strategies for individual patients.

The underlying pathophysiology is currently considered to be different between HFrEF and HFpEF (5, 6). HFrEF manifests as an eccentric remodeling accompanied with chamber dilatation and often being volume-overload leading to forward failure typically as a consequence of myocardial infarction. HFpEF is a type of concentric remodeling and/or ventricular hypertrophy characterized by impaired ventricular relaxation and/or filling, resulting in increased filling pressure and usually leading to backward failure. Recent evidences suggest that the mutual effect of cardiovascular and non-cardiovascular comorbidities [e.g., obesity (7), hypertension (8), diabetes (9), coronary artery disease (10), and chronic kidney disease (11)] induces an inflammatory state, leading to myocardial structural and functional alterations in patients with HF. The guidelines of the European Society of Cardiology (ESC) (2) and the American Heart Association (AHA) (3) suggest that the incorporation of biomarkers with clinical and imaging tools can be beneficial for establishing the diagnosis and assessing disease severity in heart failure, including biomarkers of brain-type natriuretic peptide (BNP), N-terminal pro-BNP (NT-proBNP), and cardiac troponin. Other diagnostic biomarkers, such as soluble suppression of tumorigenicity 2 (sST2), galectin-3, and growth differentiation factor-15 (GDF-15), could be beneficial in guiding HF therapy. However, the majority of the clinical biomarker data have been derived from studies in undifferentiated HF or HFrEF, while valuable prognostic biomarkers in patients with HFpEF are still very limited. Currently, there are emerging studies increasingly focusing on HFpEF which reported that strategies based on multi-biomarker and supervised/unsupervised machine learning models could improve risk stratification and prognostic prediction in HFpEF patients (12–15); however, most of them focused on traditional biomarkers, and more accurate risk stratification strategies are still needed.

In this study, we looked at 18 biomarkers which cover the main pathophysiological domains of HF, have been reported to be associated with heart failure prognosis, and can be accurately quantified in more than 95% of samples. Also, the regents with high sensitivity for testing these biomarkers are currently available in the Chinese markets. Our objectives were to assess the prognostic value of the candidate biomarkers from HF pathophysiologic pathways for 2-year all-cause mortality in patients with acute HFpEF; and establish multi-biomarker risk prediction models based on machine learning for 2-year all-cause death and cardiovascular (CV) death in patients with acute HFpEF.

## Methods

### Study Design and Patients

The current analysis included patients enrolled from the China Patient-centered Evaluative Assessment of Cardiac Events Prospective Heart Failure Study (China PEACE 5p-HF Study) between August 1, 2016 and July 31, 2017, with LVEF > 50% according to echocardiography of the standard procedure. The design of China PEACE 5p-HF Study has been described previously (16). In brief, it is a large multi-center prospective study that consecutively recruited patients hospitalized for HF between August 2016 and May 2018 from 52 hospitals (48 tertiary and 4 secondary hospitals) across China. One of the specific aims of the prospective cohort study was to identify the predictors of adverse outcomes. Patients were eligible if they were ≥18-years of age, local residents, and hospitalized with a primary diagnosis of new-onset HF or decompensation of chronic HF. Enrolled patients were interviewed during index hospitalization and followed-up at 1, 6, 12 months after discharge, and annually.

The central ethics committee at Fuwai Hospital and local internal ethics committees at study hospitals have approved the China PEACE prospective HF study. All participants provided written informed consents. The study was registered on clinicaltrials.gov (NCT 02878811).

### Data Collection

Medical history, clinical characteristics on admission, and treatments (during index hospitalization and at discharge) were centrally abstracted from medical records, with a 2-level quality control approach. In-person interviews with a standardized questionnaire during index hospitalization and follow-up were conducted to collect additional patient characteristics and outcomes. Data were directly entered into laptop computers equipped with customized electronic data collection system, allowing real-time monitoring to verify the accuracy and completeness of entered data.

### Biomarker Measurement

Blood samples were required to be obtained within 48 h after admission; and centrifuged, divided into aliquots and frozen within 1 h following the collection. Blood samples were centrifuged at 1,300 g for 10 min. Circulating levels of total cholesterol, low density lipoprotein cholesterol, high density lipoprotein cholesterol, triglycerides, and high-sensitivity C-reactive protein (hs-CRP) were measured in the serum via standardized enzymatic methods using the Beckman Coulter AU680 analyzers and Beckman AU reagent. NT-proBNP and high-sensitivity cardiac troponin T (hs-cTnT) were measured by a high-sensitivity electrochemiluminescence immunoassay on a cobas e601 analyzer with EDTA plasma. Hemoglobin A1c (HbA1c) was measured by high-performance liquid chromatography on the Arkray ADAMS-A1C HA-8180 analyzer. Circulating levels of other biomarkers were measured in the serum using a high-sensitivity Luminex Bead-Based mltiplex assay (Millopore, Billerca, MA, USA) according to the manufacturer's manual, including Endoglin, soluble tumor necrosis factor-receptor 1(sTNFRI), sTNFRII, tissue inhibitor of metalloproteinases-1 (TIMP-1), TIMP-2, matrix metalloproteinase-2 (MMP-2), MMP-8, MMP-9, galectin-3, monocyte chemoattractant protein-1(MCP-1), tumor necrosis factor (TNF)-a, GDF-15, Lipocanlin-2, Cystatin C, and sST2 (R&D Systems, Minneapolis, MN, USA).

All commercial kits were undergone internal validation prior to sample analysis. Inter/intra coefficient variation of assays was used to evaluate the assay performance. Notably, inter/intra coefficient variation of assays showed NT-proBNP <3.90%, Hs-TNT <3.40%, Hs-CRP <4.06%, GDF-15 <7.16%, MCP-1 <5.35%, TNFα <5.33%, Stnfri <6.25%, sTNFRII <6.37%, Endoglin <12.3%, TIMP-1 <6.37%, TIMP-2 <7.24%, MMP-2 <9.22%, MMP-8 <15.38%, MMP-9 <7.69%, Galectin-3 <10.53%, sST2 <7.04%, Lipocanlin-2 <5.55%, and Cystatin-C <6.62%. The assay range and inter/intra coefficient variation for per analyte were shown in Supplementary Table 1.

### Clinical Variables

Coronary heart disease (CHD), myocardial infarction (MI), valvular heart disease (VHD), atrial fibrillation, hypertension, chronic obstructive pulmonary disease (COPD), and ischemic stroke during admission were defined according to the diagnosis in medical records. Diabetes mellitus was defined according to the diagnosis in medical records or positive laboratory test results (HbA1c ≥6.5%). Reduced renal function was defined as an estimated glomerular filtration rate (eGFR) <60 mL/min/1.73 m^2^. The Acute Study of Clinical Effectiveness of Nesiritide in Decompensated Heart Failure (ASCEND-HF) outcome model was used as a reference model for predicting long-term mortality risk in patients with acute decompensated HF. ASCEND-HF outcome model is a simplified prediction model, which includes 5 commonly available clinical variables (age, dyspnea, blood urea nitrogen, sodium, and systolic blood pressure), and has a relatively good prognostic value for mortality within 30 and 180 days (17).

### Clinical Outcome

The outcomes of this study were all-cause death and CV death within 2-years after hospitalization. CV death included sudden cardiac death, death due to HF, and other CV deaths (cerebrovascular events, acute myocardial infarction, aortic vascular disease, peripheral arterial disease, and pulmonary heart disease). We ascertained outcome events with the approach employed in international multi-center clinical trials (18). Local site staffs sought information on pre-specified clinical events during follow-up interviews. If in-person follow-up visits were not feasible, information would be gathered through telephone interviews with patients, their relatives, or physicians. We also collected the information on death from the national cause-of-death database. Outcome events were centrally adjudicated by trained clinicians according to standard criteria.

### Statistical Analysis

Continuous variables were summarized as median [interquartile range (IQR)] and categorical variables as frequency (percentage). Non-parametric tests (Man-whitney-U) and Chi-Square tests were used to compare patients' baseline characteristics grouped by the 2-year survival status.

We first determined the high-risk threshold for each biomarker to divide patients into high- and low-risk groups by using the maximally selected rank statistics from the “maxstat” R package (http://cran.r-project.org/web/packages/maxstat/index.html), which is an outcome-oriented method providing a value of a cutpoint that corresponds to the most significant relation with outcome. We plotted Kaplan-Meier curves to identify the differences of 2-year all-cause death in these binary biomarkers. We used three Cox proportional hazards regression models to evaluate the relationship between individual biomarkers as binary variables and the 2-year risk of all-cause death (model 1: unadjusted model; model 2: adjusting for ASCEND-HF score and history of HF; and model 3: adjusting for ASCEND-HF score, history of HF, and NT-proBNP level). The false discovery rate (FDR) <0.05 was used to identify the significant biomarkers.

We also developed a prediction model for the 2-year risk of all-cause death with multiple biomarkers based on support vector machine (SVM) (model 6), a supervised machine learning approach. First, we randomly split the study samples into two groups, training set and validation set, in the ratio of 3:2. In the training set, with 2-year death as outcome, we trained a model with 18 biomarkers (log-NT-proBNP, hs-TNT, hs-CRP, endoglin, sTNFRI, sTNFRII, TIMP-1, TIMP-2, MMP-2, MMP-8, MMP-9, galectin-3, MCP-1, TNFα, GDF-15, lipocanlin-2, Cystatin-C, sST2), history of HF, and ASCEND-HF score, using 10-fold cross-validation, classification “C-classification,” kernel “linear,” and cost 1. We obtained each patient's probability of 2-year death based on the SVM model, which was defined as the SVM risk score. In addition, another two Cox regression models (model 4 and model 5) were developed for comparing the predictive ability with the SVM model (model 6). Model 4 was only adjusted for ASCEND-HF score and history of HF. Model 5 was adjusted for ASCEND-HF score, history of HF, and the NT-proBNP level. We compared the area under receiver operating characteristic (ROC) curves of model 6 with those of model 4 and model 5, and calculated the net reclassification improvement (NRI) and integrated discrimination improvement (IDI) by survIDINRI from R package to quantify the added predictive value of 18 biomarkers in training set and validation set, respectively.

Similarly, an SVM model for 2-year risk of CV death was developed and the value of adding 18 biomarkers to the reference model was evaluated by c-statistics, NRI, and IDI.

We conducted a sensitivity analysis by firstly dividing the study samples into training set and validation set according to the date of index admission in the ratio of 3:2, and then re-developing an SVM model and two reference models for 2-year risk of all-cause death and CV death with the same method previously mentioned. We also evaluated whether the prediction models have been improved by c-statistics, NRI, and IDI in both training set and validation set.

All calculations were performed using software SAS 9.4 and R version 4.0.3 with packages “e1071” and “maxstat.” Statistical significance was defined as a 2-tailed *p* < 0.05.

## Results

### Baseline Characteristics

We included 380 patients hospitalized for HFpEF in this analysis, whose median age was 71-years (IQR 63 to 78) and 192 of whom were female (50.5%) (Table 1). CHD (54%), VHD (28.2%), cardiomyopathy (13.2%), atrial fibrillation (56.1%), hypertension (61.1%), diabetes mellitus (34.2%), COPD (25.8%), reduced renal function (38.7%), and ischemic stroke (20%) were common comorbidities. Two-thirds of the patients had a history of HF. Most patients were in New York Heart Association (NYHA) class III/IV (87.4%) with a median (IQR) LVEF of 59% (53.4, 65.0%). During the 2-year follow-up, 102 (26.8%) patients died, among whom 84 died from CV disease. Compared with those surviving during 2-year follow-up, the dead patients were older (74-years vs. 70-years, *P* = 0.005), more likely to have COPD (*p* < 0.001), and with a higher ASCEND-HF score (*p* < 0.001) and a higher the SVM risk score (*p* < 0.001) (Table 1).

**Table 1:** Baseline characteristics stratified by survival status at 2-years after index admission.

**Baseline characteristics**	**Total (%) (*N* = 380)**	**Death (*N* = 102)**	**Survival (*N* = 278)**	***p*-value**
**Demographic**				
Age, yr (median, IQR)	71 (63, 78)	74 (67, 80)	70 (61, 77)	**0.005**
Age, group				0.125
<55	48 (12.6)	8 (7.8)	40 (14.4)	
55 to 64	61 (16.1)	12 (11.8)	49 (17.6)	
65–74	123 (32.4)	37 (36.3)	86 (30.9)	
≥75	148 (39.0)	45 (44.1)	103 (37.1)	
Female, *n* (%)	192 (50.5)	47 (46.1)	145 (52.2)	0.294
**Comorbidities**, ***n*** **(%)**				
Coronary heart disease	205 (54.0)	52 (51.0)	153 (55.0)	0.482
Myocardial infarction	55 (14.5)	18 (17.7)	37 (13.3)	0.287
Valvular heart disease	107 (28.2)	35 (34.3)	72 (25.9)	0.106
Cardiomyopathy	50 (13.2)	9 (8.8)	41 (14.8)	0.130
Coronary revascularization	48 (12.6)	16 (15.7)	32 (11.5)	0.278
Atrial fibrillation	213 (56.1)	53 (52.0)	160 (57.6)	0.330
Hypertension	232 (61.1)	62 (60.8)	170 (61.2)	0.948
Diabetes mellitus	130 (34.2)	35 (34.3)	95 (34.2)	0.980
COPD	98 (25.8)	41 (40.2)	57 (20.5)	**<0.001**
Reduced renal function^**‡**^	147 (38.7)	44 (43.1)	103 (37.1)	0.280
Ischemic stroke	76 (20.0)	19 (18.6)	57 (20.5)	0.685
**History of heart failure**	252 (66.3)	70 (68.6)	182 (65.5)	0.564
**Clinical characteristics at admission**				
SBP, mmHg, median (IQR)	133 (120, 153)	134 (115, 152)	132 (120, 153)	0.368
DBP, mmHg, median (IQR)	80 (70, 90)	79 (68, 90)	80 (70, 90)	0.093
HR, beats/min, median (IQR)	87 (74, 100)	88 (75, 101)	86 (72, 100)	0.713
NYHA functional class, *n* (%)				0.676
II	48 (12.6)	12 (11.8)	36 (13.0)	
III	182 (47.9)	46 (45.1)	136 (48.9)	
IV	150 (39.5)	44 (43.1)	106 (38.1)	
LVEF (%)	59 (53,65)	59 (54,67)	58 (53,65)	0.343
**Cardiovascular death**	84 (22.1)	84 (22.1)	NA	
**ASCEND-HF score**	5 (4, 6)	5 (4, 6)	5 (4, 5)	**<0.001**
**SVM risk score** ^*^				
Median (IQR)	0.20 (0.14, 0.29)	0.29 (0.22, 0.37)	0.18 (0.13, 0.23)	**<0.001**

*IQR, interquartile range; COPD, chronic obstructive pulmonary disease; SBP, systolic blood pressure; DBP, diastolic blood pressure; NYHA, New York Heart Association; LVEF, left ventricle ejection fraction; Hs-cTnT, high sensitivity cardiac troponin T; NT-proBNP, N-terminal brain natriuretic peptide precursor*.

‡ *Reduced renal function was defined as an estimated glomerular filtration rate (eGFR) <60 mL/min/1.73 m^2^*;

* *SVM risk score: the score is a number from 0 to 1 calculated based on the model of Support Vector Machine (SVM). P value < 0.05 is shown in bold*.

### Baseline Biomarker Levels

Table 2 shows the high-risk threshold for each biomarker and percentage of high-risk patients by individual markers at baseline in death, and survival groups. We carried out multiple comparisons with FDR analysis. The percentages of high-risk patients in the death group were significantly higher than those in the survival group for NT-proBNP (FDR <0.001), hs-TNT (FDR <0.001), hs-CRP (FDR = 0.007), GDF-15 (FDR <0.001), MCP-1 (FDR = 0.042), sTNFRI (FDR = 0.013), sTNFRII (FDR = 0.013), endoglin (FDR = 0.013), TIMP-1 (FDR <0.001), TIMP-2 (FDR <0.027), MMP-2 (FDR = 0.006), MMP-9 (FDR = 0.013), galectin-3 (FDR = 0.004), sST2 (FDR = 0.032) and Ascend-HF score (FDR <0.001) (Table 2).

**Table 2:** Percentage of high-risk patients by individual markers at baseline in the total population, death, and survival groups.

**Markers**	**Threshold (high risk)**	**Percentage in death group *n* (%)**	**Percentage in survival group *n* (%)**	***p*-value**	**FDR**
**Cardiac stretch**					
NT-proBNP	>8.0 pg/mL	50 (49.0)	46 (16.6)	**<0.001**	**<0.001**
**Cardiomyocyte injury**					
Hs-TnT, *N* (%)	>13.3 ng/L	91 (89.2)	186 (66.9)	**<0.001**	**<0.001**
**Inflammation**					
Hs-CRP	>3.7 mg/L	73 (71.6)	152 (54.7)	**0.003**	**0.007**
GDF-15	>6.9 ng/mL	29 (28.4)	28 (10.1)	**<0.001**	**<0.001**
MCP-1	<445.6 pg/mL	47 (46.1)	95 (34.2)	**0.034**	**0.042**
TNFα	>28.2 pg/mL	70 (68.6)	169 (60.8)	0.161	0.161
sTNFRI	>2.17 ng/mL	53 (52.0)	102 (36.7)	**0.007**	**0.013**
sTNFRII	>14.9 ng/mL	33 (32.4)	54 (19.4)	**0.008**	**0.013**
**Endothelial function**					
Endoglin	>3.21 ng/mL	46 (45.1)	85 (30.6)	**0.008**	**0.013**
**Extracellular matrix turnover**					
TIMP-1	>72.0 ng/mL	99 (97.1)	229 (82.4)	**<0.001**	**<0.001**
TIMP-2	>44.5 ng/mL	92 (90.2)	222 (79.9)	**0.018**	**0.027**
MMP-2	>290.7 ng/mL	39 (38.2)	63 (22.7)	**0.002**	**0.006**
MMP-8	<11.8 ng/mL	93 (91.2)	234 (84.2)	0.081	0.085
MMP-9	>133.5 ng/mL	81 (79.4)	180 (64.8)	**0.006**	**0.013**
**Fibrosis**					
Galectin-3	>9.26 ng/mL	84 (82.4)	181 (65.1)	**0.001**	**0.004**
sST2	>39.1 ng/mL	21 (20.6)	32 (11.5)	**0.025**	**0.032**
**Renal function**					
Lipocanlin-2	>289.9 ng/mL	58 (56.9)	126 (45.3)	**0.046**	0.055
Cystatin-C	>1,953 ng/mL	55 (53.9)	121 (43.5)	0.072	0.08
**Ascend_HF score**	>5.0	76 (74.5)	146 (52.5)	**<0.001**	**<0.001**

*NT-proBNP, N-terminal pro B-type brain-type natriuretic peptide; Hs-TNT, high-sensitivity cardiac troponin T; Hs-CRP, high-sensitivity C-reactive protein; GDF-15, growth differentiation factor-15; MCP-1, monocyte chemoattractant protein-1; TNFα, tumor necrosis factor-α; sTNFR, soluble tumor necrosis factor-receptor; TIMP, tissue inhibitor of metalloproteinases; MMP, matrix metalloproteinase; sST2, soluble suppression of tumorigenicity 2; P value <0.05 and FDR <0.05 are shown in bold*.

### All-Cause Death Within 2-Years of Admission

**Figure 1:**
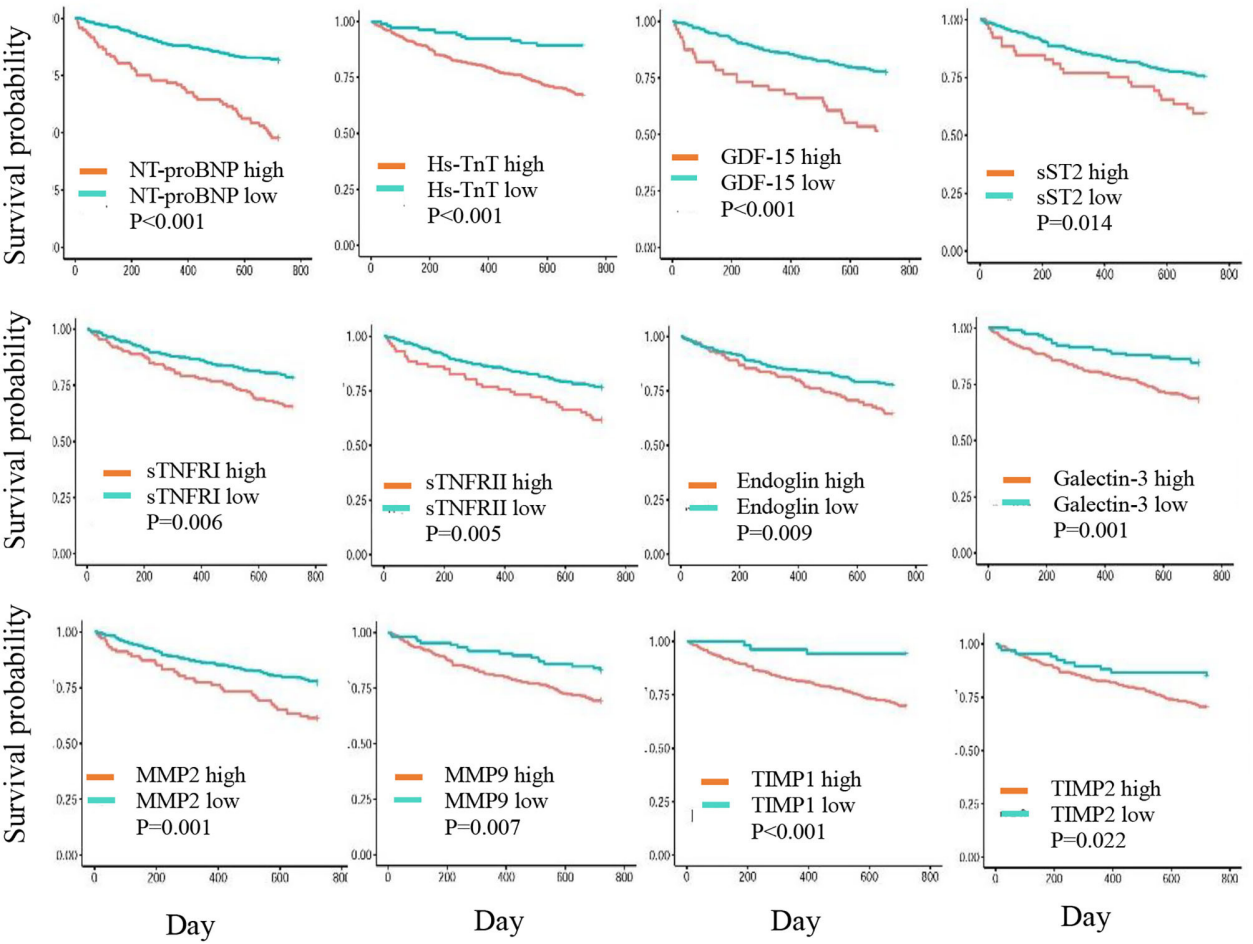
Kaplan-Meier curves showing 2-year cumulative survival trends in patients with biomarker levels in high- and low-risk groups.

In the Kaplan-Meier plots (Figure 1), patients in the high-risk group had a higher mortality rate than those in the low-risk group for the following biomarkers: log-NT-proBNP (*p* < 0.001), hs-TnT (*p* < 0.001), GDF-15 (*p* < 0.001), sTNFRI (*p* = 0.006), sTNFRII (*p* = 0.005), endoglin (*p* = 0.009), MMP2 (*p* = 0.001), MMP9 (*p* = 0.073), TIMP1 (*p* < 0.001), TIMP2 (*p* = 0.022), Galectin-3 (*p* = 0.001), and sST2 (*p* = 0.014).

### Cox Proportional Hazards Model for 2-Year All-Cause Death

Table 3 shows the association of the individual biomarkers with the 2-year risk of all-cause death in 3 Cox proportional hazards regression models. In model 3, patients in the high-risk group had a significantly increased risk of all-cause mortality compared with those in the low-risk group for multiple biomarkers, including hs-TnT, 2 inflammation-related biomarkers (GDF-15 and TNF-α), a marker of endothelial function (endoglin), and 3 biomarkers related to extracellular matrix turnover (TIMP-1, MMP-2, and MMP-9) (FDR <0.05). In model 2, hs-CRPs, TNFRII, and Galectin-3 predicted the risk of 2-year death (FDR <0.05); however, they were not significantly associated with the outcome after additional adjustment for NT-proBNP in model 3. In addition, the patients with a higher SVM risk score were associated with an increased 2-year risk of all-cause death (HR 1.80, 95% CI 1.58, 2.05), which means that the risk of mortality increased 80% with each 0.1 unit increase in the SVM risk score (Table 3).

**Table 3:** Associations between biomarkers and the 2-year risk of all-cause death by univariate and multi-variate analysis.

**Variable**	Model 1^*^ HR (95% CI)	***p*-value**	**FDR**	Model 2^*^ HR (95%CI)	***p*-value**	**FDR**	Model 3^*^ HR (95%CI)	***p*-value**	**FDR**
**Cardiac stretch**									
NT-proBNP^#^, pg/mL	3.54 (2.40–5.22)	**<0.001**	**<0.001**	3.15 (2.11–4.69)	**<0.001**	**<0.001**	NA	NA	NA
**Cardiomyocyte injury**									
Hs-TNT, ng/L	3.48 (1.86–6.50)	**<0.001**	**<0.001**	3.15 (1.67–5.94)	**<0.001**	**0.002**	2.42 (1.26–4.68)	**0.008**	**0.029**
**Inflammation**									
Hs-CRP, mg/L	1.94 (1.26–2.98)	**0.003**	**0.006**	1.94 (1.26–3.00)	**0.003**	**0.012**	1.59 (1.02–2.49)	**0.041**	0.078
GDF-15, ng/mL	2.76 (1.80–4.25)	**<0.001**	**<0.001**	2.78 (1.80–4.29)	**<0.001**	**<0.001**	2.05 (1.26–3.33)	**0.004**	**0.028**
MCP-1, pg/mL	1.50 (1.02–2.22)	**0.041**	0.051	1.46 (0.99–2.15)	0.059	0.082	1.41 (0.95–2.09)	0.086	0.146
TNFα, pg/mL	1.32 (0.87–2.00)	0.195	0.195	1.40 (0.92–2.12)	0.120	0.135	1.91 (1.22–3.00)	**0.005**	**0.028**
sTNFRI, ng/mL	1.69 (1.15–2.50)	**0.008**	**0.014**	1.47 (0.99–2.17)	0.058	0.082	1.13 (0.74–1.73)	0.584	0.662
sTNFRII, ng/mL	1.78 (1.17–2.69)	**0.007**	**0.013**	1.61 (1.05–2.45)	**0.028**	**0.049**	1.08 (0.67–1.74)	0.760	0.781
**Endothelial function**									
Endoglin, ng/mL	1.65 (1.12–2.44)	**0.012**	**0.018**	1.57 (1.06–2.34)	**0.024**	**0.049**	1.65 (1.11–2.46)	**0.013**	**0.032**
**Extracellular matrix turnover**									
TIMP-1, ng/mL	6.00 (1.90–18.9)	**0.002**	**0.006**	5.30 (1.67–16.8)	**0.005**	**0.016**	4.70 (1.48–14.9)	**0.009**	**0.029**
TIMP-2, ng/mL	2.06 (1.07–3.97)	**0.030**	**0.039**	1.97 (1.03–3.80)	**0.042**	0.069	1.70 (0.88–3.29)	0.115	0.163
MMP-2, ng/mL	1.87 (1.25–2.78)	**0.002**	**0.006**	1.75 (1.17–2.61)	**0.007**	**0.017**	1.66 (1.11–2.48)	**0.013**	**0.032**
MMP-8, ng/mL	1.76 (0.89–3.49)	0.105	0.110	1.86 (0.94–3.71)	0.077	0.099	2.05 (1.03–4.09)	**0.042**	0.078
MMP-9, ng/mL	1.91 (1.18–3.09)	**0.008**	**0.014**	1.98 (1.22–3.19)	**0.006**	**0.017**	2.05 (1.26–3.32)	**0.004**	**0.028**
**Fibrosis**									
Galectin-3, ng/mL	2.25 (1.35–3.75)	**0.002**	**0.006**	1.89 (1.12–3.18)	**0.016**	**0.036**	1.54 (0.91–2.62)	0.111	0.163
sST2, ng/mL	1.76 (1.09–2.85)	**0.021**	**0.029**	1.44 (0.88–2.36)	0.150	0.159	1.29 (0.78–2.12)	0.325	0.395
**Renal function**									
Lipocanlin-2, ng/mL	1.45 (0.98–2.15)	0.062	0.073	1.40 (0.94–2.07)	0.094	0.113	1.23 (0.82–1.83)	0.310	0.395
Cystatin-C, ng/mL	1.40 (0.95–2.07)	0.089	0.099	1.22 (0.82–1.82)	0.324	0.324	0.94 (0.62–1.43)	0.780	0.780
**ASCEND-HF score**	2.31 (1.48–3.61)	**<0.001**	**<0.001**	NA	NA	NA	NA	NA	NA
**SVM risk score** ^†^	1.80 (1.58–2.05)	**<0.001**	**<0.001**	NA	NA	NA	NA	NA	NA

* *Model 1: no adjustment; Model 2: adjusted for ASCEND-HF score and history of HF; Model 3: adjusted for ASCEND-HF score, history of HF and NT-proBNP level*.

# *The results of NT-proBNP were log-transformed for Cox proportional hazards regression models*.

† *SVM (support vector machine) risk score was used as a continuous variable. HR = 1.80 means that the risk of mortality increase 80% with each 0.1 unit increase in the SVM risk score. NT-proBNP, N-terminal pro B-type brain-type natriuretic peptide; Hs-TNT, high-sensitivity cardiac troponin T; Hs-CRP, high-sensitivity C-reactive protein; GDF-15, growth differentiation factor-15; MCP-1, monocyte chemoattractant protein-1; TNFα, tumor necrosis factor-α; sTNFR, soluble tumor necrosis factor-receptor; TIMP, tissue inhibitor of metalloproteinases; MMP, matrix metalloproteinase; sST2, soluble suppression of tumorigenicity 2,. FDR, false discovery rate. P value < 0.05 and FDR < 0.05 are shown in bold*.

### Risk Prediction Model Based on Multiple Marker Panels

**Figure 2:**
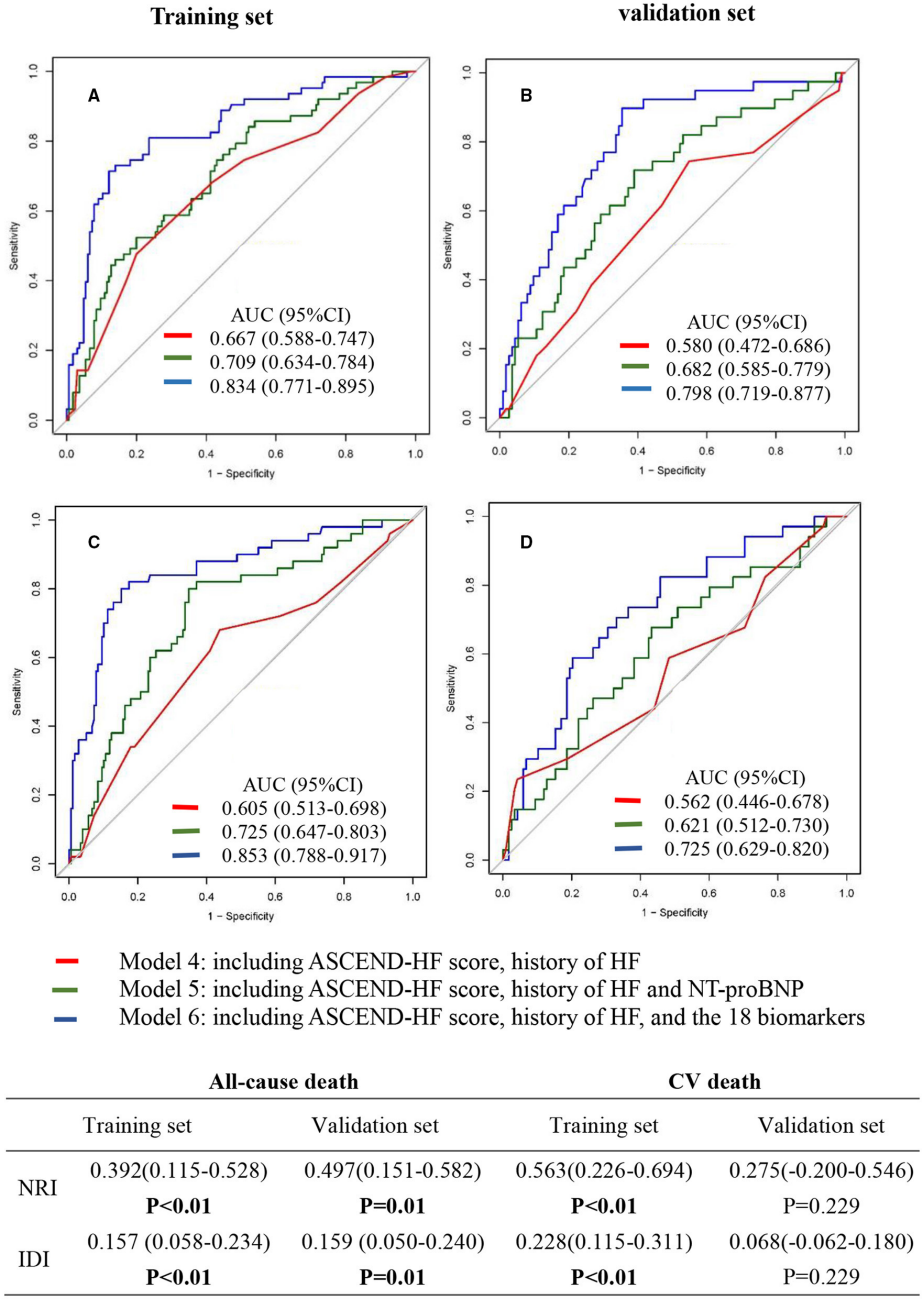
Receiver operating characteristic (ROC) curve of multi-marker models for predicting the 2-year risk of all-cause death **(A,B)** and cardiovascular death **(C,D)**. Model 4 included ASCEND-HF score and history of HF. Model 5 included ASCEND-HF score, history of HF, and NT-proBNP. Model 6 included ASCEND-HF score, history of HF, and 18 candidate biomarkers (log-NT-proBNP, hs-TNT, hs-CRP, Endoglin, sTNFRI, sTNFRII, TIMP-1, TIMP-2, MMP-2, MMP-8, MMP-9, Galectin-3, MCP-1, TNFα, GDF-15, Lipocanlin-2, Cystatin-C, sST2). NRI, net reclassification improvement; IDI, integrated discrimination improvement.

We developed 3 prediction models (model 4, model 5, and model 6) for all-cause death and CV death using different marker panels in the training set and validation set, respectively (Figure 2). All markers were used as categorical variables in these models. For all-cause death models, ROC analysis showed that model 6 (the SVM model) (AUC 0.834, 95% CI: 0.771–0.895) in the training set had better predictive effect than model 4 (AUC 0.667, 95% CI: 0.588–0.747) and model 5 (AUC 0.709, 95% CI: 0.634–0.784) (Figure 2A). The prediction ability of model 6 was improved significantly compared to model 4, with NRI 0.392 (95%CI: 0.115–0.528; *p* < 0.01) and IDI 0.157 (95%CI: 0.058–0.234; *p* < 0.01). In the validation set (Figure 2B), we also found a similar trend that model 6 (AUC 0.798, 95% CI: 0.719–0.877) showed better predictive capacity compared with model 4 (AUC 0.580, 95% CI: 0.472–0.686) and model 5 (AUC 0.682, 95% CI: 0.585–0.779). The predicted ability of model 6 was also improved significantly, with NRI 0.497 (95% CI: 0.151–0.582; *p* = 0.01) and IDI 0.159 (95% CI: 0.050–0.240; *p* = 0.01).

For CV death models, ROC analysis showed that model 6 (AUC 0.853, 95% CI: 0.788–0.917) in the training set (Figure 2C) had better predictive effect than model 4 (AUC 0.605, 95% CI: 0.513–0.698) and model 5 (AUC 0.725, 95% CI: 0.647–0.803). The predicted ability was improved significantly, with NRI 0.563 (95% CI:0.226–0.694; *p* < 0.01) and IDI 0.228 (95%CI: 0.115–0.311; *p* < 0.01). In the validation set (Figure 2D), ROC analysis also showed that model 6 (AUC 0.725, 95% CI: 0.629–0.820) had better predictive effect than model 4 (AUC 0.562, 95% CI: 0.446–0.678) and model 5 (AUC 0.621, 95% CI: 0.512–0.730). The NRI (0.275: 95% CI: −0.200 to 0.546; *p* = 0.229) and IDI (0.068: 95% CI: −0.062 to 0.180; *p* = 0.229) suggested that the improvement of the model was not statistically significant. Similar results were found in sensitivity analysis (Supplementary Figure 1).

## Discussion

In the present study, we assessed the prognostic value of circulating levels of multiple biomarkers for 2-year risk of all-cause death and CV death in patients hospitalized for HFpEF. In Cox proportional hazards models, we found that NT-proBNP (cardiac stretch biomarkers), hs-TnT (cardiomyocyte injury biomarker), 2 inflammation-related biomarkers (TNFα and GDF-15), endoglin, an endothelial function biomarker, and 3 biomarkers of extracellular matrix turnover (TIMP-1, MMP2, and MMP9) were independently associated with 2-year risk of all-cause death. We also developed prediction models of 2-year risk of all-cause death and CV death based on 18 biomarkers, history of HF, and ASCEND-HF score by machine learning, and found that the SVM model markedly improved prediction power for 2-year risk of all-cause death in both training set and validation set. It is a potentially effective approach to improve risk prediction in HFpEF patients and provide insights into the possible pathogenesis for the progression of HFpEF.

In this study, we identified an association between the endothelial dysfunction marker endoglin and 2-year risk of all-cause death, which was independent of ASCEND-HF score, history of HF, and NT-proBNP. To the best of our knowledge, our study is the first to report the independently predictive value of the biomarker for long-term risk of death in patients with HFpEF. Endoglin (also known as CD105) is a membrane co-receptor for transforming growth factor-β, which is released into the circulation in a soluble form and disrupts TGFβ1 signaling in the endothelium, thereby promoting inflammation, endothelial dysfunction, cardiac fibrosis, and vascular remodeling (19). Circulating levels of soluble endoglin were reported to elevate in patients with increased left heart filling pressures and decrease in association with reduced cardiac filling pressure after diuresis (20). Plasma endoglin has also been reported as a predictor of cardiovascular events following percutaneous coronary intervention in patients with chronic coronary artery disease (21). The elevated level of endoglin during the acute phase initially maintains cardiac output and hemodynamics in the circulation; however, it may also reflect the severity of cardiac impairment. Cardiac function deteriorates progressively when these compensatory mechanisms eventually fail over time. This may be a reason that the biomarker can predict long-term risk of death.

We also identified multiple markers of extracellular matrix turnover that were independently associated with the 2-year risk of death, including TIMP-1, MMP-2, and MMP-9; especially, TIMP1 showed the strongest association with the risk of death. In a cross-sectional study of 275 hypertensive patients, HFpEF was associated with an increased matrix turnover signal (MMP2 and MMP9). Alterations in MMP9 and TIMP1 enzymes were found to be significant indicators of greater degrees of asymptomatic left ventricular diastolic dysfunction (22). Similarly, Zile et al. reported a distinguishing role of a plasma multi-biomarker panel consisting of increased MMP-2, TIMP-4, and PIIINP and decreased MMP-8 in identifying patients with HFpEF vs. LV hypertrophy (23). Our results extend the literature with showing that abnormal extracellular matrix turnover, which plays a pivotal role in structural and functional alterations, is associated with long-term risk of death of HFpEF.

In our study, GDF-15, Gal-3, and sST2 were also found to predict the 2-year risk of death in patients with HFpEF. The results are consistent with previous studies, although the associations were attenuated after adjusting for ASCEND-HF score, history of HF, and NT-proBNP. GDF-15 is a member of the transforming growth factor-β cytokine superfamily and its expression is increased upon cell injury and inflammation. Several studies reported that GDF-15 was an independent predictor for long-term death (24) and the composite outcome of death or HF re-hospitalization in patients with HFpEF (25). Galectin-3 is a marker associated with inflammation and fibrosis. Serum levels of galectin-3 have been found to be elevated in both acute and chronic HFpEF, and they have been related to 1-year and 5-year all-cause mortality (26). sST2 is a marker associated with inflammation, myocyte hypertrophy, and fibrosis. Elevated plasma levels of sST2 have been reported to be an independent predictor of mortality and disease progression in both acute and chronic HFpEF (27, 28). Our findings further confirmed that these biomarkers could reflect disease progression and contribute to more accurate risk stratification of HFpEF patients, especially when used in combination.

Although several biomarkers have been reported to predict the outcomes in patients with HFpEF, the predictive value of individual biomarkers is limited. Machine learning has great potential to improve predictive power by combining the information of multiple biomarkers from the main pathophysiological domains of HF. Recently, Chirinos et al. (12) evaluated the prognostic value in a supervised machine-learning–derived model which combined 49 plasma biomarkers in 379 patients with chronic HFpEF. In this case, the authors found that the model was strongly predictive of the risk of HF-related hospital admission and markedly improved the risk prediction power when combined with the MAGGIC (Meta-Analysis Global Group in Chronic Heart Failure Risk Score) risk score. In addition, several studies applied unsupervised machine learning methods to identify phenotype-based subpopulations in patients with HFpEF based on clinical, laboratory and/or cardiac ultrasound data, and assessed the differences in characteristics, outcomes, as well as the levels of circulating biomarkers between different phenogroups. Hedman et al. (13) applied model-based clustering to 32 echocardiograms and 11 clinical and laboratory variables collected in 320 HFpEF outpatients, and found that the composite end point (all-cause mortality or HF hospitalization) and 15 out of 86 plasma proteins significantly varied among 6 phenogroups. Cohen et al. (14) identified 3 HFpEF phenogroups based on 8 clinical features, and observed important differences in 10 circulating biomarkers (corrected *P* < 0.05), cardiac/arterial characteristics, and prognosis (composite of cardiovascular death, heart failure hospitalization, or aborted cardiac arrest) across the clinical HFpEF phenogroups. Woolley et al. (15) performed an unsupervised cluster analysis using 363 biomarkers from 429 patients with HFpEF and identified four distinct patient subgroups. The occurrence of death or HF hospitalization during a median follow-up of 21 months had the highest rate in cluster 4 (62.8%) and the lowest in cluster 3 (25.6%). These studies provide evidence that circulating biomarkers, combined with clinical information, can help accurately identify different phenotypes in patients with HFpEF, which may reflect different pathophysiological pathways and contribute to targeted interventions for patients.

In this study, we developed a risk prediction model combining ASCEND-HF score, history of HF, and 18 circulating biomarkers based on SVM method. This model accurately predicted the 2-year risk of all-cause death in acute patients with HFpEF, suggesting that multi-biomarker models based on machine learning is a promising strategy for improving risk stratification in HFpEF. For the CV death prediction model, we found that the addition of 18 markers significantly improved the predictive value of the SVM model by ROC analysis, NRI, and IDI in the training set. However, in the validation set, NRI and IDI showed that the improvement of the model was not statistically significant. One possible reason may be due to the small sample size with fewer CV deaths in the validation set. In addition, given that heart failure can cause systemic multi-organ ischemia and dysfunction, there may also be cardiac injury in patients who died from non-cardiac causes, which may also affect the expression levels of these markers, and thus may influence the predictive power of the model.

Regarding its practical application, this multi-biomarker prediction model is promising to be applied in future clinical practice. There are currently several analytical platforms that already can simultaneously quantify multiple protein biomarkers using a very small volume of plasma samples. Besides, in light of the rapid development and increasing accessibility of analytical techniques, muti-biomarker tests would be affordable for most patients.

### Study Strengths and Limitations

This study has several strengths. First, our data is from a prospective HFpEF cohort with clear diagnoses, comprehensive baseline data, and 2-year follow-up information. Second, we used machine learning to develop a model combining 18 biomarkers with traditional clinical indicators, which could better predict the risk of death than the models developed by traditional methods. Our study also had some limitations. Firstly, cross-validation of the developed risk model using external samples was not performed in this study; a larger, independent cohort with HFpEF is needed to verify the results. Secondly, the patients included in this study are all Chinese, which limits the generalizability of our findings. Thirdly, the ASCEND-HF outcome model with good prognostic value for 30-day and 180-day mortality may not be the most appropriate reference model for this study which looks at a 2-year follow-up. However, the established models currently could not predict a longer-term risk of death in patients with acute HF. Finally, due to the low sensitivity and limited availability of detection reagents, we did not include some interesting biomarkers in this study.

## Conclusions

Multi-biomarker models based on an appropriate machine learning method can be a powerful tool for predicting long-term risk of death in patients hospitalized for HFpEF. Our findings should be verified in future studies from other ethnics.

## Data Availability Statement

The data analyzed in this study is subject to the following licenses/restrictions: The China PEACE 5p-HF Study is a national program, and as the government policy stipulates, it is not permissible for the researchers to make the raw data publicly available at this time. Requests to access these datasets should be directed to jing.li@fwoxford.org.

## Ethics Statement

The studies involving human participants were reviewed and approved by the Central Ethics Committee at Fuwai Hospital and Local Internal Ethics Committees at Study Hospitals. The patients/participants provided their written informed consent to participate in this study.

## Author Contributions

JinL, YG, and JiaL designed the study. XB and HD designed the biostatistical methods and analyzed the data. YG drafted the manuscript. Other authors revised the manuscript for important intellectual content. All the authors participated in interpretation of the data and approved the final version of the manuscript.

## Funding

This project was supported by the National Key Technology R&D Program (2015BAI12B02) from the Ministry of Science and Technology of China, CAMS Innovation Fund for Medical Sciences (2017-I2M-2-002), and the National natural science foundation of China (81903399).

## Conflict of Interest

JinL reported receiving research grants, through Fuwai Hospital, from the Chinese government for work to improve the management of hypertension and blood lipids, to improve care quality and patient outcomes of cardiovascular disease, and to improve care for COVID-19 infection; receiving research agreements, through the National Center for Cardiovascular Diseases and Fuwai Hospital, from Amgen for a multicenter clinical trial assessing the efficacy and safety of omecamtiv mecarbil and for dyslipidemic patient registration; receiving a research agreement, through Fuwai Hospital, from Sanofi for a multicenter clinical trial on the effects of sotagliflozin; receiving a research agreement, through Fuwai Hospital, with the University of Oxford for a multicenter clinical trial of empagliflozin; receiving a research agreement, through the National Center for Cardiovascular Diseases, from AstraZeneca for clinical research methods training outside the submitted work; and receiving a research agreement, through the National Center for Cardiovascular Diseases, from Lilly for physician training outside the submitted work. The remaining authors declare that the research was conducted in the absence of any commercial or financial relationships that could be construed as a potential conflict of interest.

## Publisher's Note

All claims expressed in this article are solely those of the authors and do not necessarily represent those of their affiliated organizations, or those of the publisher, the editors and the reviewers. Any product that may be evaluated in this article, or claim that may be made by its manufacturer, is not guaranteed or endorsed by the publisher.
