# Supplementary material for: Prognostic Value of Multiple Circulating Biomarkers for 2-Year Death in Acute Heart Failure With Preserved Ejection Fraction
Source: Front Cardiovasc Med. 2021 Dec 9;8:779282. doi: 10.3389/fcvm.2021.779282 (PMC8695736; doi:10.3389/fcvm.2021.779282)
Supplement: Supplementary file 1 [file Data_Sheet_1.docx]

**Prognostic Value of Multiple Circulating Biomarkers for 2-Year Death in Acute Heart Failure with Preserved Ejection Fraction**

**Supplemental material**

**Table S1. The assay range and coefficient of variation of the 18 biomarkers**

| Biomarkers pg/mL | Lower limit of quantitation | Upper limit of quantitation | Coefficient of variation (%) |
| --- | --- | --- | --- |
| NT-proBNP | 5 | 384357 | < 3.90 |
| Hs-TNT | 3 | 34616 | < 3.40 |
| Hs-CRP | 0.16 | 41.2 | < 4.06 |
| GDF-15 | 0.31 | 8777 | < 7.16 |
| MCP-1 | 2.95 | 17234 | < 5.35 |
| TNFα | 2.92 | 18714 | < 5.33 |
| sTNFRI | 20.25 | 40909 | < 6.25 |
| sTNFRII | 16.52 | 79102 | < 6.37 |
| Endoglin | 151.02 | 19929 | < 12.30 |
| TIMP-1 | 12.38 | 13996 | < 6.37 |
| TIMP-2 | 47.31 | 55241 | < 7.24 |
| MMP-2 | 73.65 | 60193 | < 9.22 |
| MMP-8 | 174.30 | 358713 | < 15.38 |
| MMP-9 | 7.18 | 14947 | < 7.69 |
| Galectin-3 | 2.97 | 6501 | < 10.53 |
| sST2 | 358.38 | 130529 | < 7.04 |
| Lipocanlin-2 | 3.60 | 171081 | < 5.55 |
| Cystatin-C | 59.58 | 104160 | < 6.62 |

NT-proBNP, N-terminal pro B-type brain-type natriuretic peptide; Hs-TNT, high-sensitivity cardiac troponin T; Hs-CRP, high-sensitivity C-reactive protein; GDF-15, growth differentiation factor-15; MCP-1, monocyte chemoattractant protein-1; TNFα, tumor necrosis factor-α; sTNFR, soluble tumor necrosis factor-receptor; TIMP, tissue inhibitor of metalloproteinases; MMP, matrix metalloproteinase; sST2, soluble suppression of tumorigenicity 2.

**Figure S1. Receiver operating characteristic (ROC) curve of multi-marker models for predicting the 2-year risk of all-cause death (A, B) and cardiovascular death (C, D) in sensitivity analysis.** Model 4 included ASCEND-HF score and history of HF. Model 5 included ASCEND-HF score, history of HF, and NT-proBNP. Model 6 included ASCEND-HF score, history of HF, and 18 candidate biomarkers (log-NT-proBNP, hs-TNT, hs-CRP, Endoglin, sTNFRI, sTNFRII, TIMP-1, TIMP-2, MMP-2, MMP-8, MMP-9, Galectin-3, MCP-1, TNFα, GDF-15, Lipocanlin-2, Cystatin-C, sST2).

|  | **All-cause death** | | **CV death** | |  |
| --- | --- | --- | --- | --- | --- |
|  | Training set | Validation set | Training set | Validation set | |
| NRI | 0.433(0.167-0.562) **P<0.01** | 0.433(0.149-0.571) **P<0.01** | 0.410(0.058-0.585) **P=0.01** | 0.280(-0.071-0.456) P=0.139 | |
| IDI | 0.185(0.094-0.253) **P<0.01** | 0.185(0.094-0.253) **P<0.01** | 0.194(0.070-0.280) **P=0.01** | 0.098(-0.001-0.229) P=0.06 | |


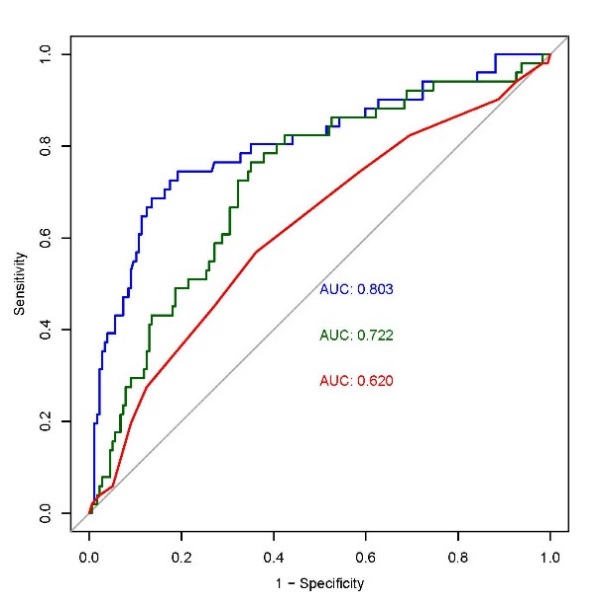

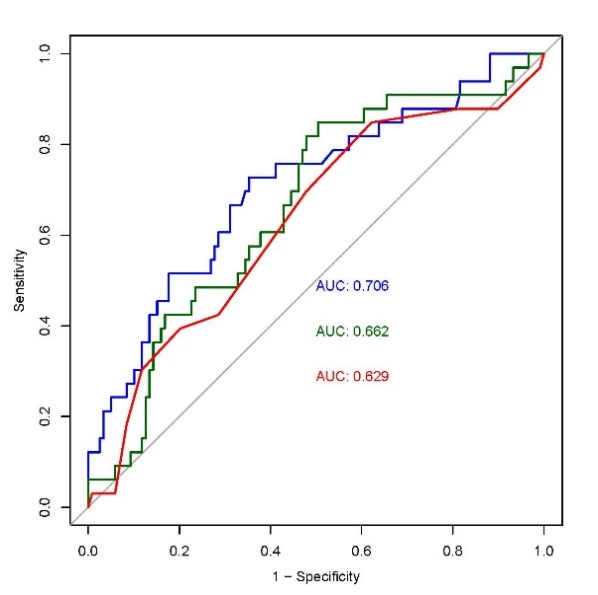


AUC (95CI)

0.620 (0.531-0.710)

0.721 (0.642-0.801)

0.803 (0.727-0.880)

AUC (95CI)

0.629 (0.519-0.740)

0.662 (0.559-0.765)

0.706 (0.601-0.811)

**A**

**B**

**C**

**D**

**Training set**

**validation set**


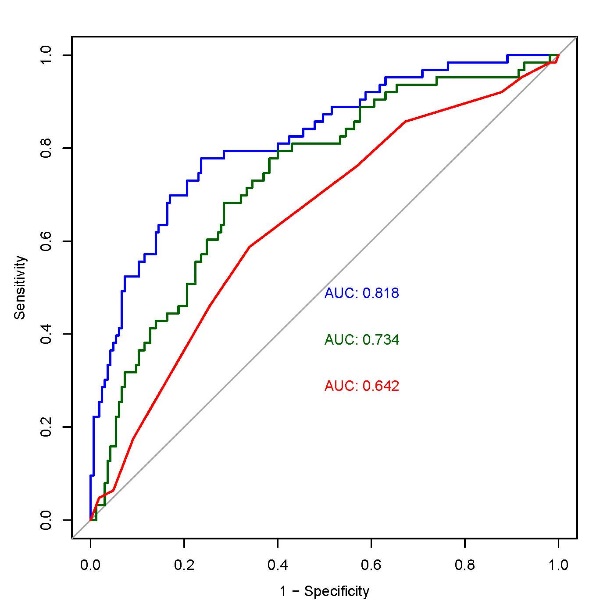


AUC (95CI)

0.642 (0.562-0.722)

0.734 (0.662-0.806)

0.818 (0.755-0.881)


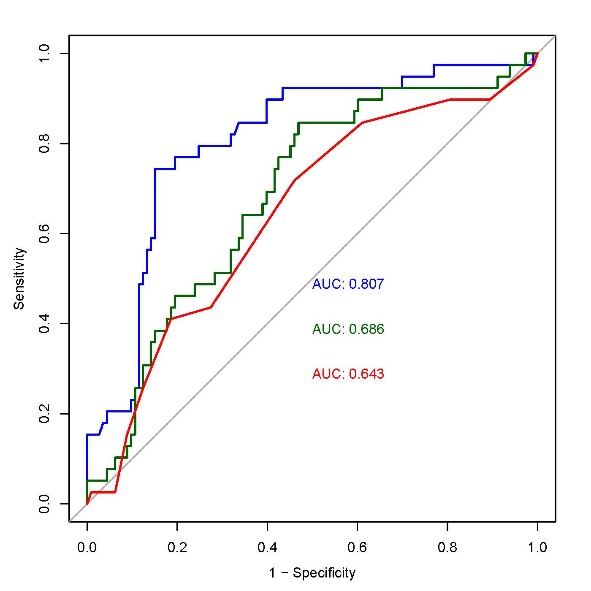


AUC (95%)

0.643 (0.542-0.744)

0.686 (0.591-0.781) 0.807 (0.726-0.888)

Model4 including ASCEND-HF score, history of HF

Model5 including ASCEND-HF score, history of HF and NT-proBNP

Model6 including ASCEND-HF score, history of HF, and the 18 biomarkers

**A**

**B**
